# Joint effects of temperature and humidity with PM_2.5_ on COPD

**DOI:** 10.1186/s12889-025-21564-3

**Published:** 2025-02-03

**Authors:** Huan Minh Tran, Feng-Jen Tsai, Yuan-Hung Wang, Kang-Yun Lee, Jer-Hwa Chang, Chi-Li Chung, Chien-Hua Tseng, Chien-Ling Su, Yuan-Chien Lin, Tzu-Tao Chen, Kuan-Yuan Chen, Shu-Chuan Ho, Feng-Ming Yang, Sheng-Ming Wu, Kian Fan Chung, Kin-Fai Ho, Kai-Jen Chuang, Hsiao-Chi Chuang

**Affiliations:** 1 College of Public Health, Program in Global Health and Health Security, Taipei Medical University, Taipei, Taiwan; 2 Faculty of Public Health, Da Nang University of Medical Technology and Pharmacy, Da Nang, Viet Nam; 3 Graduate Institute of Clinical Medicine, College of Medicine, Taipei Medical University, Taipei, Taiwan; 4 Department of Medical Research, Shuang Ho Hospital, Taipei Medical University, New Taipei City, Taiwan; 5 Division of Pulmonary Medicine, Department of Internal Medicine, Shuang Ho Hospital, Taipei Medical University, New Taipei City, Taiwan; 6 Division of Pulmonary Medicine, Department of Internal Medicine, School of Medicine, College of Medicine, Taipei Medical University, Taipei, Taiwan; 7 Inhalation Toxicology Research Lab (ITRL), School of Respiratory Therapy, College of Medicine, Taipei Medical University, 250 Wuxing Street, Taipei, 11031 Taiwan; 8 Division of Pulmonary Medicine, Department of Internal Medicine, Wan Fang Hospital, Taipei Medical University, Taipei, Taiwan; 9 Division of Pulmonary Medicine, Department of Internal Medicine, Taipei Medical University Hospital, Taipei Medical University, Taipei, Taiwan; 10 Division of Critical Care Medicine, Department of Emergency and Critical Care Medicine, Shuang Ho Hospital, Taipei Medical University, New Taipei City, Taiwan; 11 Department of Civil Engineering, National Central University, Taoyuan City, Taiwan; 12 National Heart and Lung Institute, Imperial College London, London, UK; 13 The Jockey Club School of Public Health and Primary Care, The Chinese University of Hong Kong, ShatinHong Kong, N.T China; 14 School of Public Health, College of Public Health, Taipei Medical University, Taipei, Taiwan; 15 Department of Public Health, School of Medicine, College of Medicine, Taipei Medical University, Taipei, Taiwan; 16 Cell Physiology and Molecular Image Research Center, Wan Fang Hospital, Taipei Medical University, Taipei, Taiwan; 17 School of Respiratory Therapy, College of Medicine, Taipei Medical University, Taipei, Taiwan

**Keywords:** COPD, PM_2.5_, Relative humidity, Short-term exposure, Temperature

## Abstract

**Background:**

Particulate matter less than 2.5 microns in aerodynamic diameter (PM_2.5_) is a significant air pollutant known to adversely affect respiratory health and increase the incidence of chronic obstructive pulmonary disease (COPD). Furthermore, climate change exacerbates these impacts, as extreme temperatures and relative humidity (RH) levels can intensify the effects of PM_2.5_. This study aims to examine the joint effects of PM_2.5_, temperature, and RH on the risk of COPD.

**Methods:**

A case–control study was conducted among 1,828 participants from 2017 to 2022 (995 COPD patients and 833 controls). The radial basis function interpolation was utilized to estimate participants' individual mean and differences in PM_2.5_, temperature, and RH in 1-day, 7-day, and 1-month periods. Logistic regression models examined the associations of environmental exposures with the risk of COPD adjusting for confounders. Joint effects of PM_2.5_ by quartiles of temperature and RH were also examined.

**Results:**

We observed that a 1 µg/m^3^ increase in PM_2.5_ 7-day and 1-month mean was associated with a 1.05-fold and 1.06-fold increase in OR of COPD (*p* < *0.05*). For temperature and RH, we observed U-shaped effects on OR for COPD with optimal temperatures identified as 21.2 °C, 23.8 °C, and 23.8 °C for 1-day, 7-day, and 1-month mean temperature, respectively, and optimal RH levels identified as 73.8%, 76.7%, and 75.4% for 1-day, 7-day, and 1-month mean RH, respectively (*p* < *0.05*). The joint effect models show that high temperatures (> 23.5 °C) and both extremely low (69.3%) and high (80.9%) RH levels generally exacerbate the effects of PM_2.5_ on OR for COPD, especially over longer exposure durations.

**Conclusion:**

The joint effects of PM_2.5_, temperature, and RH on the risk of COPD underscore the importance of air pollution control and comprehensive research to mitigate COPD risk in the context of climate change.

**Supplementary Information:**

The online version contains supplementary material available at 10.1186/s12889-025-21564-3.

## Background

Particulate matter less than 2.5 microns in aerodynamic diameter (PM_2.5_) is a key air pollutant known to significantly affect respiratory health and increase the incidence of chronic obstructive pulmonary disease (COPD) [1]. In 2017, the Global Burden of Disease estimated that PM_2.5_ accounted for 19.3% of all disability-adjusted life years for COPD [2]. On the other hand, temperature and relative humidity (RH), as environmental factors, may influence the extent of the impact of PM_2.5_ [3]. The joint effects of these elements under changing climatic conditions could potentially alter the risk of COPD [4].

Previous studies have demonstrated the significant impact of PM_2.5_ on the risk of COPD [5]. There is evidence that elevated PM_2.5_ levels were associated with increased COPD incidence rates [6]. For example, in a general population, the observed increase in the prevalence rates of COPD for each standard deviation increment in PM_2.5_ ranged between 1.10 and 1.25 times [6]. A previous systematic review found that a 10 µg/m^3^ daily increase in PM_2.5_ concentration was associated with a 1.60% (95% CI: 0.40–2.90%) increase in the risk of COPD hospitalization [7], while another review found that 1 µg/m^3^ increase in PM_2.5_ was associated with 1.03 (95%CI: 1.00–1.06) times higher risk of COPD [8]. Furthermore, climate change exacerbates these impacts, as extreme temperatures and altered RH levels can intensify air pollution effects [9]. Elevated temperatures often lead to increased formation of ground-level ozone, which, together with high PM_2.5_ levels, can worsen respiratory conditions [10]. For RH, a large-scale evidence from Korea’s national health surveys shows that RH exerts nuanced impacts on lung function, negatively associated with a forced expiratory volume in 1 s (FEV₁)/forced vital capacity (FVC) ratio [11]. For instance, in short-term exposures (≤ 7 days), each 1% increase in RH was associated with a β of −0.015 (*p* < *0.05*) at lag 0 day and −0.016 (*p* < *0.05*) at lag 1 day for FEV₁/FVC [11]. Complementary mechanistic insights suggest that both very low and very high humidity can disrupt airway epithelial integrity, mucociliary clearance, and immune defenses, thus influencing infection rates and allergic symptoms [12]. These findings underscore the need to incorporate RH into epidemiologic models of COPD risk.

PM_2.5_ can exacerbate respiratory conditions by penetrating deep into lung tissues, causing oxidative stress and inflammation that impair pulmonary function [1]. Additionally, meteorological factors like temperature and relative humidity may modulate the body's response to these particles [13]. For instance, higher temperatures can enhance the chemical reactions that form secondary organic aerosols, increasing the toxicity of particulate matter [14]. On the other hand, RH influences the hygroscopic growth of PM, affecting its deposition in the respiratory tract and its ability to carry soluble toxic components into the lungs [15], while independently impacting lung health by influencing airway responsiveness and facilitating the survival and transmission of respiratory pathogens, which may relate to COPD [16]. These interactions suggest a complex interplay where PM_2.5_ and meteorological factors together can exacerbate the inflammatory responses and oxidative stress, leading to respiratory diseases such as COPD.

Existing studies have focused on the individual effects of PM_2.5_, temperature, and RH on the risk of COPD diagnosis [17–19]. However, there is a critical need to understand how these factors jointly affect the risk of COPD diagnosis. This case–control study examines the joint effects of PM_2.5_, temperature, and RH on the risk of COPD diagnosis, as measured by the odds ratio (OR), thus offering vital insights into the complex environmental determinants of COPD.

## Methods

### Study population

A case–control study included 1,828 participants, including 995 COPD patients and 833 controls. COPD patients (case group) were consecutively recruited at three hospitals in Taipei from January 2017 to December 2022. On the other hand, controls were selected from the Tucheng Health Care Cohort (THCC) in New Taipei City, gathered from a health project running from October 2018 to April 2021. These patients were newly diagnosed with COPD at the time of the lung function test conducted during the study. Prior to this, they had not received a COPD diagnosis. To be defined as a COPD patient for this study, individuals had to meet the diagnostic criteria based on a FEV_1_/ FVC ratio (on pre-bronchodilator spirometry) below 70% [20], between the ages of 40 and 90, and they could not have had any exacerbations in the past three months or been diagnosed with any excluded conditions, such as cancer, bronchiectasis, asthma, or other unrelated progressive inflammatory diseases. Controls were selected from the Tucheng Health Care Cohort (THCC) in New Taipei City, derived from a health project operational from October 2018 to April 2021. Controls met similar age criteria and were excluded if they had any respiratory conditions or the aforementioned excluded conditions to ensure comparability with cases. Both groups underwent pre-bronchodilation lung function tests performed by trained healthcare technicians and nurses using the Vitalograph Spirotac VTM, adhering to the standards of the American Thoracic Society/European Respiratory Society [21]. Additionally, covariates concerning age, sex, medical history, body mass index (BMI), smoking status, and respiratory symptoms were consistently collected by practical physicians before conducting the lung function test. The Taipei Medical University-Joint Institution Review Board (TMU-JIRB no. N202302060) approved the study protocol.

### Individual-level exposure to ambient RH, temperature, and PM_2.5_

The radial basis function (RBF) interpolation was chosen to estimate ambient exposure to PM2.5, RH, and temperature for each participant based on their addresses because it produces smoother and more accurate interpolations compared to other methods, such as inverse distance weighting [22–25]. The linear kernel function was specifically used to optimize spatial estimates in the study area. Hourly data for RH and temperature were acquired from Taiwan's Central Weather Bureau, and hourly PM_2.5_ concentrations were sourced from air monitoring stations operated by Taiwan's Environmental Protection Administration. After initial organization and cleaning, these hourly PM_2.5_ data were spatially estimated for each participant and then aggregated into daily mean to reflect daily exposure levels accurately. To assess the impact of exposure over time, we calculated both the mean and difference in values of PM_2.5_, temperature, and RH across 1-day, 7-day, and 30-day intervals. The mean exposure was calculated using data from 1-day, 7-days, and 1-month prior to the lung function test, up to and including the day of the event.

The mean value of t day was calculated from the (t-1) day before the case to the case day. This can be mathematically represented as:

$$\overline{x }=\frac{{\sum }_{i=(1-t)}^{0}{x}_{i}}{t}$$

Where, 'x' is the time period, 'xi' represents for environmental exposure factors, and 't' is number days in the period. The difference value was calculated by taking the difference between the current and the previous mean values for the same period length.

$$\overline{\Delta x }=\frac{{\sum }_{i=(1-t)}^{0}{x}_{i}}{t}-\frac{{\sum }_{i=(1-2t)}^{-t}{x}_{i}}{t}$$

Detailed methodologies for calculating the mean and differences in environmental variables have been published previously [26].

### Data analysis

The Shapiro–Wilk test was used to assess the normal distribution of the FEV_1_/FVC. To mitigate the impact of outliers, extreme values beyond the 99th percentile were adjusted using the Winsorization method [27]. The association of PM_2.5_, temperature, and RH with the OR for COPD among cases (COPD patients) and controls (non-COPD individuals) was investigated using logistic regression. For sensitivity analysis, a linear regression was utilized to assess the associations of PM_2.5_, temperature, and RH with lung function measured by FEV_1_/FVC, FEV_1_, and FVC. We also explored the non-linearity in these associations and developed dose–response curves to illustrate the variations in OR for COPD across different levels of PM_2.5_, temperature, and RH. These curves were analyzed using generalized additive models (GAMLSS) with penalized splines (ps) and four degrees of freedom [28]. To further examine potential joint effects, temperature, and RH were divided into quartiles and joined into the PM_2.5_ analysis using the GAMLSS model to assess their joint effects on COPD risk [29]. The sensitivity analysis was also conducted for FEV_1_, FVC, and FEV_1_/FVC. All statistical models were adjusted for age, sex, smoking habits, and BMI. All analyses were conducted using R software version 4.2.2, with a statistical significance level set at *p* < *0.05.*

## Results

### Characteristics of study subjects

Table 1 shows the clinical characteristics of 1,828 participants, including 995 with COPD and 833 controls. The COPD group had a significantly higher proportion of males (83.7%) and current smokers (35.2%) compared to the control group, which had 22.6% males and 6.8% current smokers (*p* < *0.05*). The average age was higher in COPD patients (70.5 ± 10.7 years) than in controls (63.0 ± 8.2 years). Additionally, COPD patients had a lower BMI (23.5 ± 4.0 kg/m^2^) compared to the control group (24.6 ± 3.6 kg/m^2^) (*p* < *0.05*). Lung function, as measured by FEV_1_-pred, was lower in the COPD group, averaging 63.7 ± 21.6%, compared to 88.0 ± 15.9% in the controls, with FEV_1_/FVC at 58.5 ± 12.0% for the COPD group and 83.2 ± 6.2% for controls (*p* < *0.05*).

**Table 1:** Basic characteristics of study subjects (*N* = 1828)

Factors	COPD group (*n* = 995)	Control group (*n* = 833)	*P*-value
**Gender, n (%)**			
Males	833 (83.7)	243 (22.6)	**0.000**
Females	162 (16.3)	590 (78.4)	
**Age, Mean ± SD (Years)**	70.5 ± 10.7	63.0 ± 8.2	**0.000**
**BMI, Mean ± SD (kg/m**^**2**^**)**	23.5 ± 4.0	24.6 ± 3.6	**0.000**
**Smoking, n (%)**			
Current	350 (35.2)	57 (6.8)	**0.000**
Ex-smokers	465 (46.7)	75 (9.0)	
Non-smokers	180 (18.1)	701 (84.2)	
**FEV₁, Mean ± SD (%pred)**	63.7 ± 21.6	88.0 ± 15.9	**0.000**
**FVC, Mean ± SD (%pred)**	83.4 ± 23.0	84.8 ± 15.4	0.132
**FEV₁/FVC, Mean ± SD (%)**	58.5 ± 12.0	83.2 ± 6.2	**0.000**
**PM**_**2.5**_**, µg/m**^**3**^** (min–max)**			
1-Day mean	15.96 ± 9.3 (3.6–47.5)	15.74 ± 8.3 (1.7–60.4)	0.594
7-Day mean	13.9 ± 4.7 (5.3–35.4)	15.2 ± 5.8 (4.5–43.8)	**0.000**
1-Month mean	14.0 ± 3.7 (8.1–30.7)	15.2 ± 4.7 (6.7–36.1)	**0.000**
1-Day difference	0.9 ± 7.3 (−11.4–23.7)	0.3 ± 7.5 (−41.7–38.1)	0.131
7-Day difference	−0.2 ± 4.7 (−13.4–12.3)	0.3 ± 5.5 (−21.8–18.3)	**0.037**
1-Month difference	0.4 ± 3.1 (−8.4–7.6)	−0.1 ± 3.6 (−9.9–12.3)	**0.001**
**Temperature, °C (min–max)**			
1-Day mean	23.7 ± 4.6 (16.7–31.8)	23.4 ± 5.3 (8.1–32.4)	0.354
7-Day mean	23.2 ± 4.8 (14.6–31.1)	23.4 ± 5.0 (11.2–32.1)	0.414
1-Month mean	23.4 ± 4.8 (15.0–30.9)	23.5 ± 4.7 (13.7–31.1)	0.906
1-Day difference	0.2 ± 1.5 (−3.0–4.0)	−0.1 ± 1.9 (−9.5–4.4)	**0.006**
7-Day difference	−0.2 ± 2.1 (−4.7–5.3)	−0.1 ± 2.2 (−6.9–5.9)	0.182
1-Month difference	−0.4 ± 2.3 (−4.3–4.0)	−0.2 ± 2.8 (−7.0–5.9)	**0.022**
**RH, % (min–max)**			
1-Day mean	75.7 ± 7.8 (49.7–93.1)	74.5 ± 8.9 (47.8–98.2)	**0.002**
7-Day mean	75.3 ± 5.3 (62.8–91.2)	74.6 ± 6.7 (57.2–93.9)	**0.020**
1-Month mean	75.6 ± 3.4 (67.7–86.9)	74.5 ± 4.9 (64.8–91.4)	**0.000**
1-Day difference	0.9 ± 7.2 (−16.5–21.9)	0.2 ± 7.1 (−27.3–29.2)	**0.043**
7-Day difference	−0.3 ± 7.0 (−19.8–12.9)	−0.1 ± 6.9 (−20.0–18.3)	0.552
1-Month difference	−0.2 ± 4.3 (−11.8–7.6)	0.2 ± 5.1 (−14.5–13.8)	0.079

*COPD* Chronic Obstructive Pulmonary Disease, *BMI* body mass index, *FEV*_*1*_ post-bronchodilator forced expiratory volume in 1 s, *FVC* Forced Vital Capacity, *PM*_*2.5*_ particulate matter with an aerodynamic diameter of < 2.5 μm, *RH* relative humidity

### Temperature, RH, and PM_2.5_ exposure

Table 1 presents the environmental exposures, including PM_2.5_, temperature, and RH, between the COPD and control groups over various periods (1-day, 7-day, and 1-month). We observed that PM_2.5_ levels were significantly higher in the control group for both the 7-day and 1-month means (*p* < *0.05*). There were no significant differences in temperature between the groups during these periods. However, we found that RH was consistently higher in the COPD group across all measured periods, with the most notable difference observed in the 1-day mean RH (75.7 ± 7.8% for COPD vs. 74.5 ± 8.9% for controls; *p* < *0.05*). Table S1 indicates negative correlations between PM_2.5_, temperature, and RH, except for a positive correlation between PM_2.5_ and temperature.

### Associations of PM_2.5_, temperature, and RH with COPD risk

**Fig. 1:**
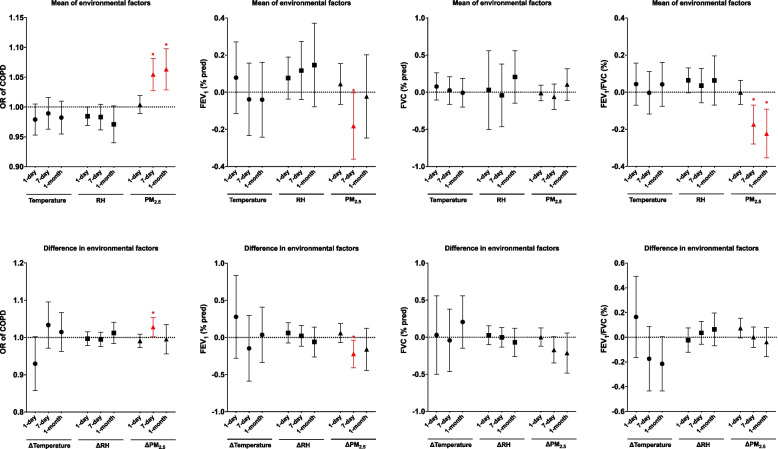
Associations of the daily mean and difference in ambient temperature, relative humidity (RH), and particulate matter with an aerodynamic diameter of < 2.5 μm (PM_2.5_) for 1-day, 7-day, and 1-month mean and difference with the odds ratio (OR) for chronic obstructive pulmonary disease (COPD), forced expiratory volume in 1 s (FEV_1_), forced vital capacity (FVC) and FEV_1_/FVC. Data are presented as the OR for COPD and FEV_1_/FVC estimates with a 95% confidence interval (CI). Covariates adjusted for in the models were age, sex, smoking, and body mass index (BMI). Red asterisk (*) indicates statistical significance at *p* < *0.05*

Figure 1 illustrates the associations of mean and differences in temperature, RH, and PM_2.5_ over 1-day, 7-day, and 1-month periods with the OR for COPD and lung function. For COPD risk, we observed that a 1 µg/m^3^ increase in the 7-day mean PM_2.5_ level was associated with a 1.05-fold increase in the OR for COPD (95% CI: 1.03–1.08; *p* < *0.05*). A 1 µg/m^3^ increase in the 1-month mean PM_2.5_ level was associated with a 1.06-fold increase in the OR for COPD (95% CI: 1.03–1.10; *p* < *0.05*). Regarding lung function, we found that a 1 µg/m^3^ increase in the 7-day mean PM_2.5_ level was associated with a 0.18% decrease in FEV₁-predicted and a 0.17% decrease in the FEV_1_/FVC ratio (95% CI: 0.06–0.28; *p* < *0.05*). A 1 µg/m^3^ increase in the 1-month mean PM_2.5_ level was associated with a 0.22% decrease in the FEV_1_/FVC ratio (95% CI: 0.09–0.35; *p* < *0.05*). Additionally, a 1 µg/m^3^ increase in the 7-day difference in PM_2.5_ levels was associated with a 1.03-fold increase in the OR for COPD (95% CI: 1.00–1.05; *p* < *0.05*), a 0.22% decrease in FEV_1_-predicted, and a 0.17% decrease in the FEV_1_/FVC ratio (95% CI: 0.04–0.41; *p* < *0.05*).

### Non-linear effects of PM_2.5_, temperature, and RH on COPD risk

**Fig. 2:**
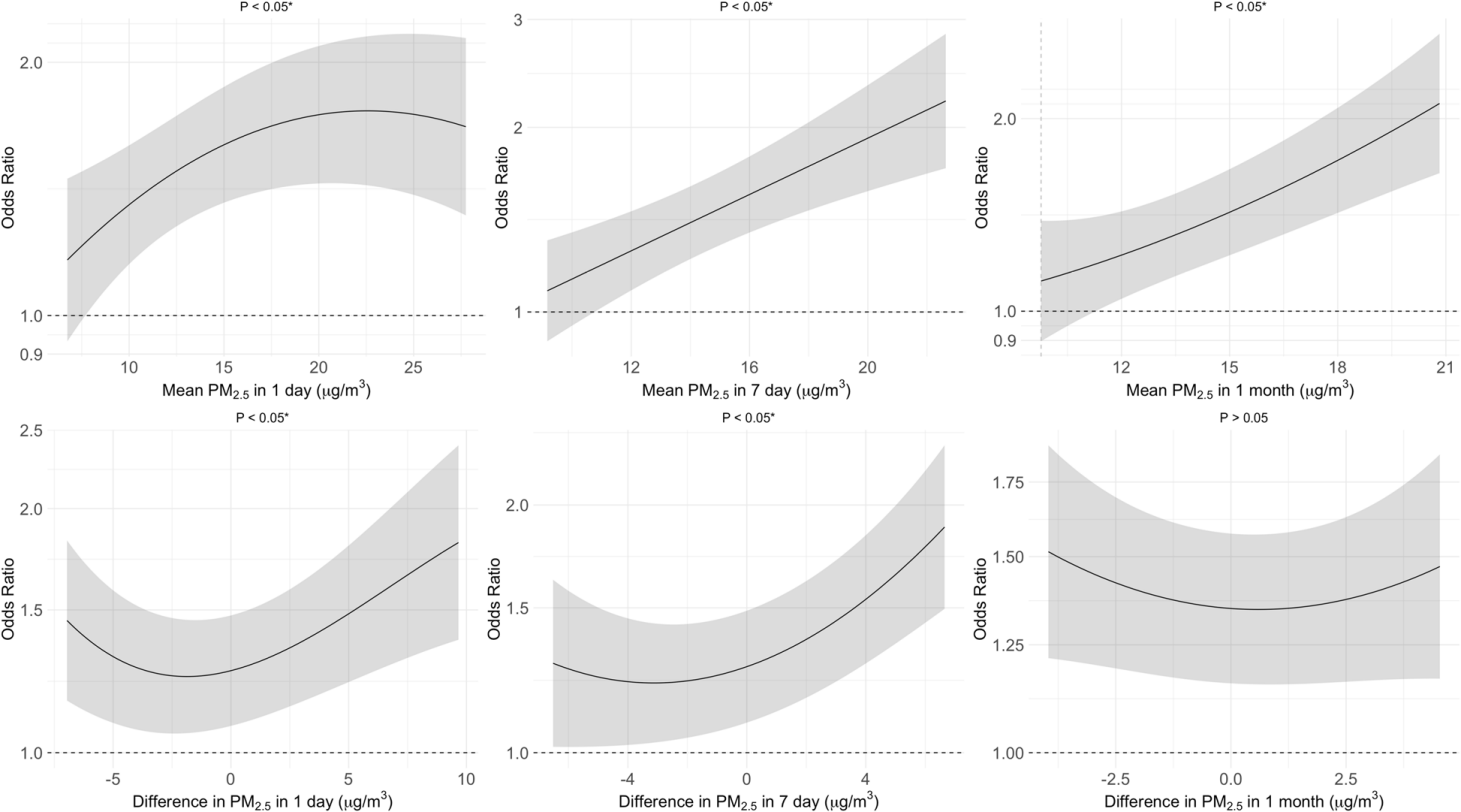
Exposure–response relationship between the particulate matter with an aerodynamic diameter of < 2.5 μm (PM_2.5_) and the odds ratio (OR) for chronic obstructive pulmonary disease (COPD). The solid line represents the OR for COPD, given the daily mean and difference in PM_2.5_ exposure. The shaded area shows the 95% CI, reflecting the precision of the OR estimate. Covariates adjusted for in the models were age, sex, smoking, and body mass index (BMI). The numbers in blue denote the OR at specific PM_2.5_ levels, with the corresponding red numbers indicating the PM_2.5_ concentration. Asterisks (*) mark statistically significant associations where *p* < 0.05. The dashed green line across the OR of 1.0 serves as a reference

**Fig. 3:**
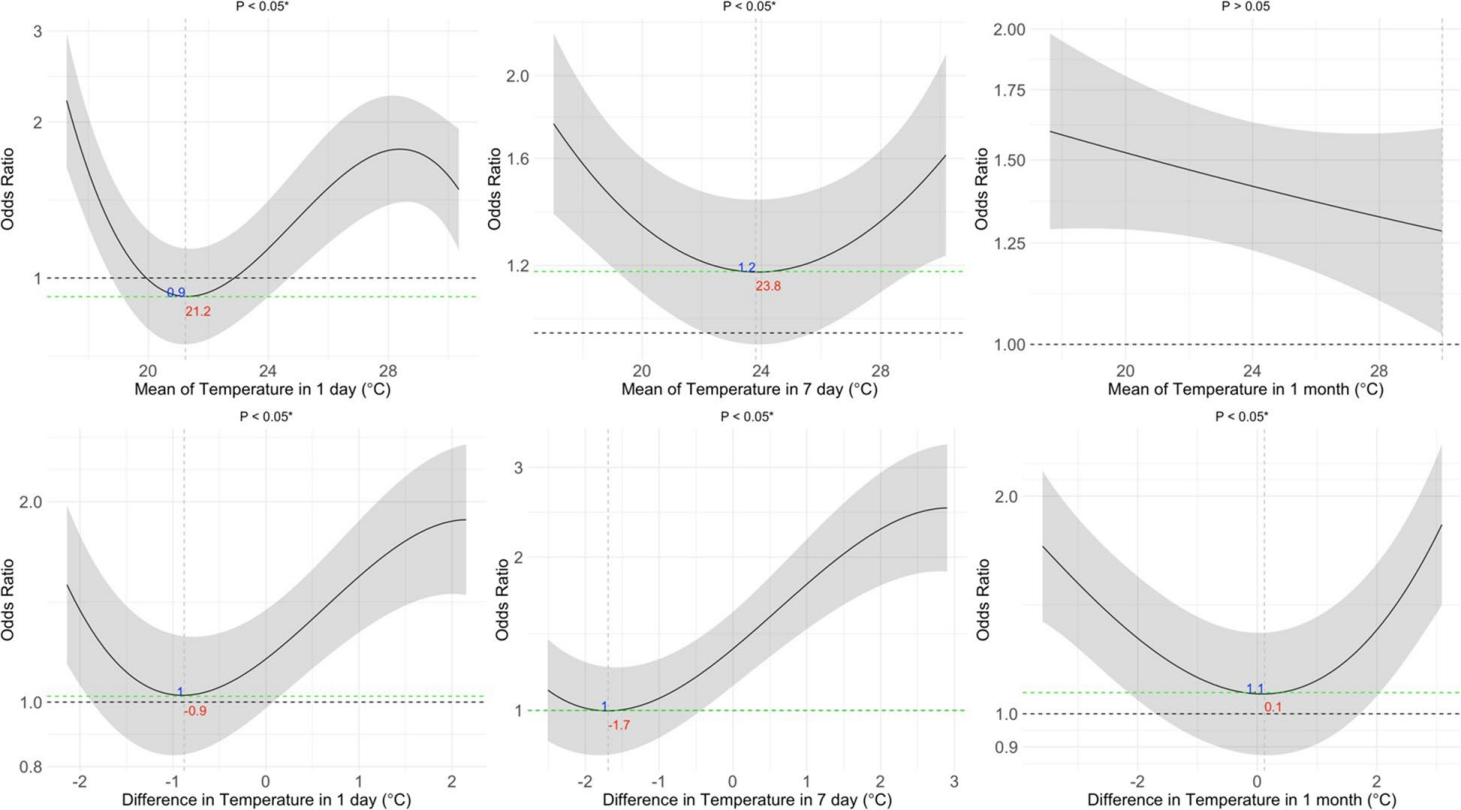
Exposure–response relationship between the temperature and the risk of chronic obstructive pulmonary disease (COPD). The solid line represents the odds ratio (OR) for COPD, given the daily mean and difference in temperature exposure. The shaded area shows the 95% CI, reflecting the precision of the OR estimate. Covariates adjusted for in the models were age, sex, smoking, and body mass index (BMI). The numbers in blue denote the OR at specific temperature levels, with the corresponding red numbers indicating the temperature concentration. Asterisks (*) mark statistically significant associations where *p* < *0.05*. The dashed green line across the OR of 1.0 serves as a reference

**Fig. 4:**
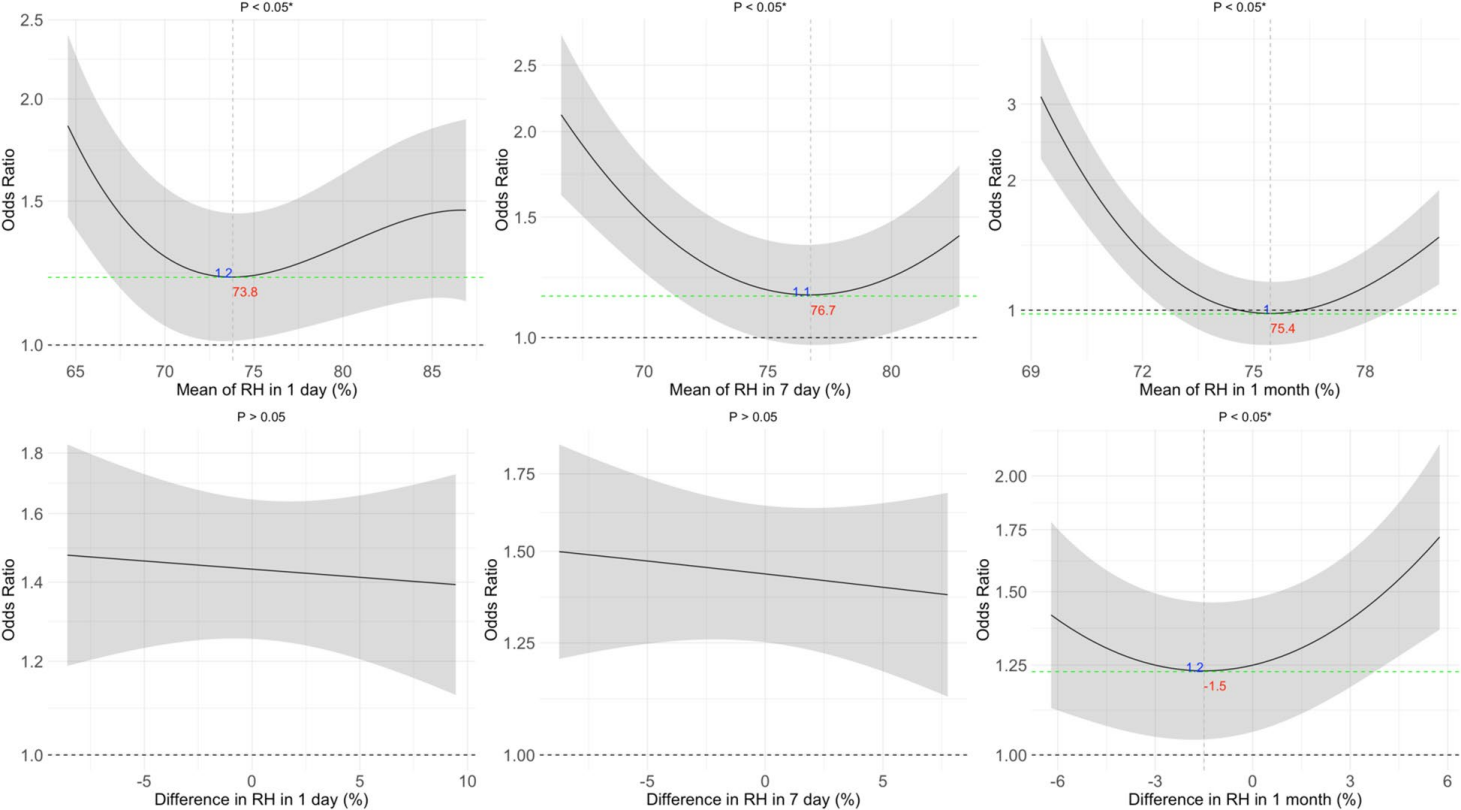
Exposure–response relationship between the relative humidity (RH) and the risk of chronic obstructive pulmonary disease (COPD). The solid line represents the odds ratio (OR) for COPD, given the daily mean and difference in RH exposure. The shaded area shows the 95% CI, reflecting the precision of the OR estimate. Covariates adjusted for in the models were age, sex, smoking, and body mass index (BMI). The numbers in blue denote the OR at specific temperature levels, with the corresponding red numbers indicating the temperature concentration. Asterisks (*) mark statistically significant associations where *p* < *0.05*. The dashed green line across the OR of 1.0 serves as a reference

Figures 2, 3 and 4 illustrate the non-linear associations between PM_2.5_, temperature, and RH exposures with the OR for COPD. We observed that there were significant non-linear effects of the 1-day, 7-day, and 1-month mean PM_2.5_ levels on the OR for COPD, as well as for the 1-day and 7-day differences in PM_2.5_ (*p* < *0.05*). For temperature, we observed U-shaped effects of the 1-day, 7-day, and 1-month mean temperatures on the OR for COPD, with optimal temperatures identified as 21.2 °C for the 1-day mean, 23.8 °C for both the 7-day and 1-month means. We also found non-linear associations for the 1-day and 7-day differences in temperature, highlighting optimal differences of −0.9 °C and −1.7 °C, respectively (*p* < *0.05*). For RH, we observed U-shaped effects of the 1-day, 7-day, and 1-month mean RH on the OR for COPD, with optimal RH levels identified as 73.8% for the 1-day mean, 76.7% for the 7-day mean, and 75.4% for the 1-month mean. We observed a non-linear association for the 1-month difference in RH, highlighting an optimal difference of −1.5% (*p* < *0.05*). Figures S1-S3 show that these non-linear effects were more pronounced among males than females. For sensitivity analysis, Figures S4–S12 illustrate the non-linear associations between PM_2.5_, temperature, and RH with FEV_1_/FVC, FEV_1_, and FVC. We observed that PM_2.5_ exposure was associated with declines in FEV₁/FVC and FEV₁, temperature exposure was associated with variations in FEV₁/FVC, and RH was associated with FEV₁/FVC (*p* < *0.05*).

### Joint effects of PM_2.5_ and temperature on the risk of COPD

**Fig. 5:**
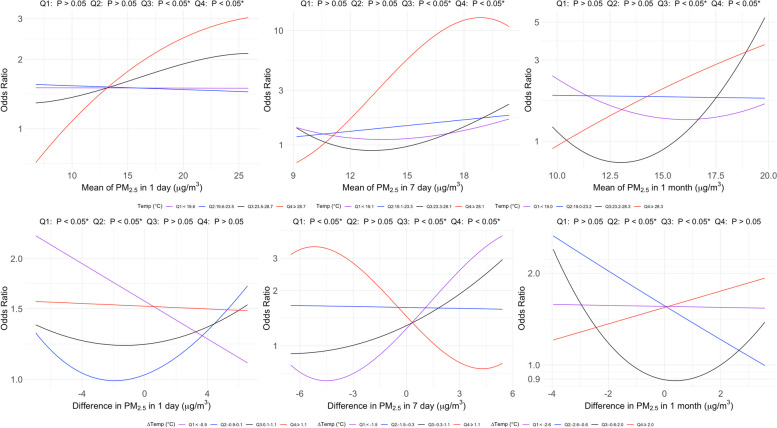
Associations of odds ratio (OR) for chronic obstructive pulmonary disease (COPD) with particulate matter of an aerodynamic diameter of < 2.5 μm (PM_2.5_) were analyzed, considering the joint effect by quartiles of temperature. Two-factor models were fitted for the OR for COPD, with four degrees of freedom restricted, and adjusted for age, sex, body mass index, and smoking status. The temperature was divided into quartiles: first quartile (Q_1_), second quartile (Q_2_), third quartile (Q_3_), and fourth quartile (Q_4_), and joined into the PM_2.5_ analysis using the GAMLSS model to assess their joint effects on COPD risk. Asterisks (*) mark statistically significant associations where *p* < *0.05*

Figure 5 demonstrates the joint effects of PM_2.5_ and temperature quartiles on the OR for COPD. We observed significant joint effects in certain quartiles, particularly in Q3 (23.5–28.7 °C) and Q4 (≥ 28.7 °C). These findings indicate complex joint effects between PM_2.5_ exposure and temperature on the OR for COPD (*p* < *0.05*). The curves highlight that extremely high temperatures generally exacerbated the effects of PM_2.5_ on OR for COPD, especially over longer exposure durations. Figures S13 and S14 show that these joint effects were more pronounced among males than females. For sensitivity analysis, Figures S15–S17 show the joint effects of PM_2.5_ and temperature quartiles on lung function, with significant joint effects in Q3 (23.5–28.7 °C) and Q4 (≥ 28.7 °C).

### Joint effects of RH and PM_2.5_ on the risk of COPD

**Fig. 6:**
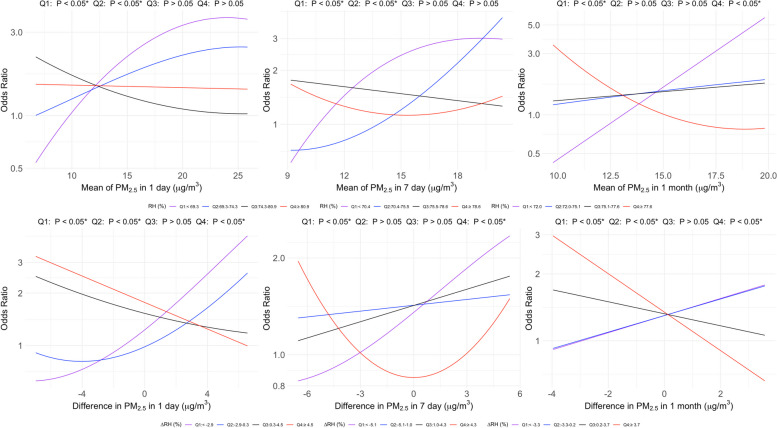
Associations of odds ratio (OR) of chronic obstructive pulmonary disease (COPD)with particulate matter of an aerodynamic diameter of < 2.5 μm (PM_2.5_) were analyzed, considering the joint effect by quartiles of relative humidity (RH). Two-factor models were fitted for the OR for COPD, with four degrees of freedom restricted, and adjusted for age, sex, body mass index, and smoking status. The RH was divided into quartiles: first quartile (Q_1_), second quartile (Q_2_), third quartile (Q_3_), and fourth quartile (Q_4_), and joined into the PM_2.5_ analysis using the GAMLSS model to assess their joint effects on COPD risk. Asterisks (*) mark statistically significant associations where *p* < *0.0*

Figure 6 illustrates the joint effects of PM_2.5_ and RH quartiles on the OR for COPD. We observed significant joint effects in some quartiles, particularly in Q1 (≤ 69.3%) and Q4 (≥ 80.9%). These results indicate complex joint effects between PM_2.5_ exposure and RH on the OR for COPD (*p* < *0.05*). The curves suggest that extreme RH levels exacerbated the impact of PM_2.5_ on OR for COPD, especially over longer exposure durations. Figures S18 and S19 show significant interactions in Q1 (≤ 69.3%) and Q4 (≥ 80.9%) for both males and females. Figures S20–S22 illustrate the sensitivity analysis of the joint effects of PM_2.5_ and RH quartiles on lung function, showing that extreme RH levels exacerbated the impact of PM_2.5_ on lung function.

## Discussion

In the context of a rapidly changing climate and increasing air pollution, our study evaluates the joint effects of environmental factors on the risk for COPD. While COPD is a chronic disease typically developing over prolonged exposure to factors like smoking and long-term air pollution, our study utilizes short-term exposure data to identify significant associations between PM₂.₅, temperature, RH, and COPD risk. The novelty of our study lies in identifying that higher temperatures and both low and high humidity, in conjunction with PM_2.5_, decrease lung function and increase the OR for COPD. These associations may reflect acute effects on lung function declines, underscoring the importance of considering environmental factors in COPD management even over shorter time frames. Our findings highlight PM_2.5_'s critical role in respiratory health and the need for a comprehensive strategy to address environmental factors influencing COPD risk.

Although the control group had higher mean PM_2.5_ levels due to individual exposure variability, logistic regression analysis demonstrated that increased PM₂.₅ exposure wass associated with higher risk of COPD. The linear association of PM_2.5_ with COPD has been shown in previous studies [30]. A study conducted in Thailand revealed that each 1% rise in PM_2.5_ concentrations corresponded to a 0.25% increase in newly diagnosed cases of COPD [19]. A previous investigation involving 96,779 individuals revealed that for every increase of 10 μg/m^3^ in PM_2.5_ levels at the residence of a participant, there was an elevated risk for COPD, with an OR of 1.55 [29]. The mechanism underlying this positive association may involve PM_2.5_ exposure leading to the generation of reactive oxygen species and inflammation in the respiratory system, which in turn contributes to the development of COPD [31, 32].

Next, we showed the U-shaped associations of temperature and RH with the increasing risk of COPD and decreased lung function. This study shows that both low and high temperatures are linked to COPD risk, with lower temperatures having a notably stronger effect. Prior research indicates a potential threshold (18 °C) below which lower temperatures could negatively affect respiratory diseases [33]. Cold exposure can cause unique respiratory reactions like runny nose, congestion, damage to the airway lining resulting in structural and functional changes, and bronchoconstriction due to cooling of facial skin and upper airways [34]. Additionally, some studies have noted an increase in hospital admissions due to COPD on days marked by very high temperatures [35]. Research conducted in New York City revealed a 7.6% rise in COPD hospital admissions for each 1 °C rise in temperature above a critical threshold of 29 °C [36]. Another study encompassing 12.5 million older adults across 213 urban counties in the U.S. found a 4.7% higher risk of COPD-related hospital admissions with each 10-degree Fahrenheit rise in ambient temperature [35]. The detrimental impact of extreme temperatures likely stems from their role in heightening the risk of respiratory infections and impairing lung function [37–39]. RH was found to have a U-shaped association with lung function declines and the risk for COPD, especially low RH. In line with our results, the study in Hong Kong showed a hockey-stick pattern with the lowest point of RH at 82% was associated with increased cases of COPD [40]. Low humidity can lead to bronchoconstriction and dry out the mucosal lining of the airway, heightening the risk of infections [41]. Conversely, extreme RH was also associated with an increased risk for COPD, as extreme humidity levels can foster environments conducive to the growth of dust mites and bacteria, potentially aggravating COPD symptoms [42]. Meanwhile, a previous review indicates that when RH deviates too far from the ~ 40–60% range, it can compromise airway epithelial integrity, potentially doubling viral infectivity at < 40% RH or enabling fungal overgrowth at > 60% RH, which may increase the risk of respiratory diseases such as COPD [12]. Together, extremely high and low temperatures and RH were associated with a higher risk for COPD.

Finally, we observed that higher temperatures and both low and high RH, in joint effects with PM_2.5_, increase the risk for COPD. First, the joint effects of high temperatures and PM_2.5_ lead to decreased lung function and, subsequently, a higher risk for COPD. Previous research showed that high temperatures and PM_2.5_ exposure synergistically worsen lung function, potentially leading to COPD [43], especially in older adults [44]. This joint effect exacerbates respiratory inflammation and impairs lung cleansing, hindering the body's ability to remove harmful particles [45]. Second, low humidity levels can cause the airways to dry and become irritated, making them more susceptible to damage from inhaled pollutants like PM_2.5_ [12]. Dry air can also impair the mucociliary clearance process, which increases vulnerability to infections and inflammation, contributing to COPD risk [46]. Third, high humidity amplifies air pollution's effects, like PM_2.5_, on lung function, potentially causing COPD. A study found a 5% rise in RH 4 h before examination linked to a 0.3% FVC decrease (95% CI:0.1–0.5, *p* < *0.05*) and a 0.2% FEV_1_ decrease (95% CI:0.0–0.4, *p* < *0.05*) in populations exposed to high levels of black carbon [44]. It can be explained that when extreme RH is joined with PM_2.5_, which itself can carry toxic substances and pathogens, the risk of respiratory infections and inflammation increases, potentially decreasing lung function and leading to COPD [47]. Together, the joint effects of high temperature and both low and high RH with PM_2.5_ decrease lung function and increase the risk for COPD.

Our study, investigating the joint effects of temperature and humidity on associations of PM_2.5_ and COPD, faces limitations such as bias from predominantly male smokers, excluding factors like indoor air pollution and job-related exposures, and using hospital data that might not be widely applicable due to selection bias. Although this study utilizes extensive hospital and cohort data, its non-randomized design, uneven numbers of cases and controls, and lack of individual matching by age and sex may limit the generalizability of findings and introduce residual confounding despite statistical adjustments. Additionally, because other pollutants (e.g., PM_10_, NO_2_, O_3_) were not fully controlled in the final models, ascertaining the independent effect of PM_2.5_ remains challenging. Future research should account for other pollutants such as PM_10_, NO_2_, O_3_, indoor air quality, workplace exposures, and long-term exposure assessments over periods of 1 year or more to fully capture the chronic aspects of climate change and PM_2.5_ exposure to COPD risk. Future prospective cohort studies with extended follow-up should incorporate biological markers of airway inflammation and oxidative stress and utilize Distributed Lag Nonlinear Models (DLNM) to establish causal relationships and accurately assess the delayed and cumulative effects of long-term exposures to air pollution, temperature, and humidity on COPD development.

## Conclusions

Our findings highlight an association between increased levels of PM_2.5_ in conjunction with high temperature and both low and high RH in increasing COPD risk. By aligning with WHO guidelines, it is recommended that COPD patients regularly exposed to PM_2.5_, extreme temperatures, and humidity monitor environmental conditions and adopt protective strategies. These strategies include limiting outdoor activities during periods of high pollution or extreme weather, using air purifiers and masks, and maintaining optimal indoor temperature and humidity levels to mitigate associated risks. Together, these approaches can effectively address the growing challenge of COPD in the context of global climate change and air pollution.

## Supplementary Information

Supplementary Material 1.

## Data Availability

Data will be made available on request.

## Abbreviations

BMI: Body mass index
COPD: Chronic obstructive pulmonary disease
CI: Confidence interval
FEV_1_: Forced expiratory volume in 1 s
FVC: Forced vital capacity
OR: Odds ratio
PM_2.5_: Particulate matter less than 2.5 microns in aerodynamic diameter
RH: Relative humidity
TMU-JIRB: Taipei Medical University-Joint Institution Review Board
THCC: Tucheng Health Care Cohort
