# Supplementary material for: Joint effects of temperature and humidity with PM2.5 on COPD
Source: BMC Public Health. 2025 Feb 3;25:424. doi: 10.1186/s12889-025-21564-3 (PMC11789386; doi:10.1186/s12889-025-21564-3)
Supplement: Supplementary file 1 — Supplementary Material 1. [file 12889_2025_21564_MOESM1_ESM.docx]

**SUPPLEMENTARY INFORMATION**

**Joint Effects of Temperature and Humidity with PM_2.5_ on COPD**

Huan Minh Tran^1,2#^ (MD.), Feng-Jen Tsai^1#^ (PhD), Yuan-Hung Wang^3,4#^ (PhD.), Kang-Yun Lee^5,6^ (MD, PhD.), Jer-Hwa Chang^7,8^ (MD, MSc), Chi-Li Chung^7,9^ (MD, PhD.), Chien-Hua Tseng^5,6,10^ (MD, PhD.), Chien-Ling Su^5,7^ (PhD.), Yuan-Chien Lin^11^ (PhD.), Tzu-Tao Chen^3,5,6^ (MD), Kuan-Yuan Chen^3,5^ (MD), Shu-Chuan Ho^5,7^ (PhD.), Feng-Ming Yang^7^ (PhD.), Sheng-Ming Wu^5,6^ (PhD.), Kian Fan Chung^12^ (MD., DSc.), Kin-Fai Ho^13^ (PhD.), Kai-Jen Chuang^14,15^ (PhD.), Hsiao-Chi Chuang^5,7,12,16*^ (PhD.)

*^1^Ph.D. Program in Global Health and Health Security, College of Public Health, Taipei Medical University, Taipei, Taiwan*

*^2^Faculty of Public Health, Da Nang University of Medical Technology and Pharmacy, Da Nang, Viet Nam*

*^3^Graduate Institute of Clinical Medicine, College of Medicine, Taipei Medical University, Taipei, Taiwan*

*^4^Department of Medical Research, Shuang Ho Hospital, Taipei Medical University, New Taipei City, Taiwan*

*^5^Division of Pulmonary Medicine, Department of Internal Medicine, Shuang Ho Hospital, Taipei Medical University, New Taipei City, Taiwan*

*^6^Division of Pulmonary Medicine, Department of Internal Medicine, School of Medicine, College of Medicine, Taipei Medical University, Taipei, Taiwan*

*^7^School of Respiratory Therapy, College of Medicine, Taipei Medical University, Taipei, Taiwan*

*^8^Division of Pulmonary Medicine, Department of Internal Medicine, Wan Fang Hospital, Taipei Medical University, Taipei, Taiwan*

*^9^ Division of Pulmonary Medicine, Department of Internal Medicine, Taipei Medical University Hospital, Taipei Medical University, Taipei, Taiwan*

*^10^Division of Critical Care Medicine, Department of Emergency and Critical Care Medicine, Shuang Ho Hospital, Taipei Medical University, New Taipei City, Taiwan*

*^11^Department of Civil Engineering, National Central University, Taoyuan City, Taiwan*

*^12^National Heart and Lung Institute, Imperial College London, London, UK*

*^13^The Jockey Club School of Public Health and Primary Care, The Chinese University of Hong Kong, Shatin, N.T., Hong Kong, China*

*^14^School of Public Health, College of Public Health, Taipei Medical University, Taipei, Taiwan*

*^15^Department of Public Health, School of Medicine, College of Medicine, Taipei Medical University, Taipei, Taiwan*

*^16^Cell Physiology and Molecular Image Research Center, Wan Fang Hospital, Taipei Medical University, Taipei, Taiwan*

**Running head:** Climate increase impact of PM_2.5_ on COPD

**^#^These authors contributed equally to this work**

**Corresponding Authors**

*Hsiao-Chi Chuang, PhD*

Inhalation Toxicology Research Lab (ITRL), School of Respiratory Therapy, College of Medicine, Taipei Medical University, 250 Wuxing Street, Taipei 11031, Taiwan.

Telephone: +886-2-27361661 ext. 3513. Fax: +886-2-27391143. E-mail: chuanghc@tmu.edu.tw

**Table S1.** Correlation between ambient relative humidity (RH), temperatures, and PM_2.5_ in daily mean and difference for 1-day, 7-day, and 1-month averages for study subjects (N = 1828)

|  | **1-day RH** | **7-day RH** | **1-month RH** | **1-day Temperature** | **7-day Temperature** | **1-month Temperature** | **1-day PM_2.5_** | **7-day PM_2.5_** | **1-month PM_2.5_** |
| --- | --- | --- | --- | --- | --- | --- | --- | --- | --- |
| **1-day RH** |  | -0.043 | -0.083* | -0.297* | 0.069* | -0.067* | 0.054* | -0.002 | 0.050* |
| **7-day RH** | 0.625* |  | -0.024 | -0.093* | -0.199* | 0.077* | -0.002 | -0.231* | -0.129* |
| **1-month RH** | 0.402* | 0.624* |  | -0.146* | -0.120* | -0.337* | -0.060* | 0.040 | -0.331* |
| **1-day Temperature** | -0.314* | -0.401* | -0.563* |  | 0.008 | 0.042 | 0.110* | 0.015 | 0.121* |
| **7-day Temperature** | -0.219* | -0.356* | -0.596* | 0.923* |  | 0.138* | -0.097* | 0.319* | 0.050* |
| **1-month Temperature** | -0.177* | -0.296* | -0.582* | 0.871* | 0.951* |  | -0.013 | 0.017 | -0.139* |
| **1-day PM_2.5_** | -0.117* | -0.041 | 0.017 | -0.094* | -0.213* | -0.292* |  | -0.189* | 0.041 |
| **7-day PM_2.5_** | -0.178* | -0.233* | -0.069* | -0.111* | -0.181* | -0.312* | 0.623* |  | 0.050* |
| **1-month PM_2.5_** | -0.134* | -0.128* | -0.094* | -0.206* | -0.266* | -0.386* | 0.540* | 0.792* |  |

RH, relative humidity; PM_2.5_, particulate matter with an aerodynamic diameter of <2.5 μm; orange indicates the mean values, blue indicates the different values

**Figure S1.** Exposure-response relationship between the PM_2.5_ and the OR for COPD stratified by gender. The solid line represents the OR for COPD, given the daily mean and difference in PM_2.5_ exposure. The shaded area shows the 95% CI, reflecting the precision of the OR estimate. Covariates adjusted for in the models were age, sex, smoking, and body mass index (BMI). Asterisks (*) mark statistically significant associations where *p<0.05*.

**Figure S2.** Exposure-response relationship between the temperature and the OR for COPD stratified by gender. The solid line represents the OR for COPD, given the daily mean and difference in temperature exposure. The shaded area shows the 95% CI, reflecting the precision of the OR estimate. Covariates adjusted for in the models were age, sex, smoking, and body mass index (BMI). Asterisks (*) mark statistically significant associations where *p<0.05*

**Figure S3.** Exposure-response relationship between the RH and the OR for COPD stratified by gender. The solid line represents the OR for COPD, given the daily mean and difference in RH exposure. The shaded area shows the 95% CI, reflecting the precision of the OR estimate. Covariates adjusted for in the models were age, sex, smoking, and body mass index (BMI). Asterisks (*) mark statistically significant associations where *p<0.05*

**Figure S4.** Exposure-response relationship between the PM_2.5_ and the FEV_1_/FVC. The solid line represents the FEV_1_/FVC, given the daily mean and difference in PM_2.5_ exposure. The shaded area shows the 95% CI, reflecting the precision of the FEV_1_/FVC estimate. Covariates adjusted for in the models were age, sex, smoking, and body mass index (BMI). Asterisks (*) mark statistically significant associations where *p<0.05*

**Figure S5.** Exposure-response relationship between the temperature and the FEV_1_/FVC. The solid line represents the FEV_1_/FVC, given the daily mean and difference in temperature exposure. The shaded area shows the 95% CI, reflecting the precision of the FEV_1_/FVC estimate. Covariates adjusted for in the models were age, sex, smoking, and body mass index (BMI). Asterisks (*) mark statistically significant associations where *p<0.05*
**Figure S6.** Exposure-response relationship between the RH and the FEV_1_/FVC. The solid line represents the FEV_1_/FVC, given the daily mean and difference in RH exposure. The shaded area shows the 95% CI, reflecting the precision of the FEV_1_/FVC estimate. Covariates adjusted for in the models were age, sex, smoking, and body mass index (BMI). Asterisks (*) mark statistically significant associations where *p<0.05*

**Figure S7.** Exposure-response relationship between the PM_2.5_ and the FEV_1_. The solid line represents the FEV_1_, given the daily mean and difference in PM_2.5_ exposure. The shaded area shows the 95% CI, reflecting the precision of the FEV_1_ estimate. Covariates adjusted for in the models were age, sex, smoking, and body mass index (BMI). Asterisks (*) mark statistically significant associations where *p<0.05*

**Figure S8.** Exposure-response relationship between the Temperature and the FEV_1_. The solid line represents the FEV_1_, given the daily mean and difference in Temperature exposure. The shaded area shows the 95% CI, reflecting the precision of the FEV_1_ estimate. Covariates adjusted for in the models were age, sex, smoking, and body mass index (BMI). Asterisks (*) mark statistically significant associations where *p<0.05*

**Figure S9.** Exposure-response relationship between the RH and the FEV_1_. The solid line represents the FEV_1_, given the daily mean and difference in RH exposure. The shaded area shows the 95% CI, reflecting the precision of the FEV_1_ estimate. Covariates adjusted for in the models were age, sex, smoking, and body mass index (BMI). Asterisks (*) mark statistically significant associations where *p<0.05*

**Figure S10.** Exposure-response relationship between the PM_2.5_ and the FVC. The solid line represents the FVC, given the daily mean and difference in PM_2.5_ exposure. The shaded area shows the 95% CI, reflecting the precision of the FEV_1_ estimate. Covariates adjusted for in the models were age, sex, smoking, and body mass index (BMI). Asterisks (*) mark statistically significant associations where *p<0.05*

**Figure S11.** Exposure-response relationship between the temperature and the FVC. The solid line represents the FVC, given the daily mean and difference in temperature exposure. The shaded area shows the 95% CI, reflecting the precision of the FEV_1_ estimate. Covariates adjusted for in the models were age, sex, smoking, and body mass index (BMI). Asterisks (*) mark statistically significant associations where *p<0.05*

**Figure S12.** Exposure-response relationship between the RH and the FVC. The solid line represents the FVC, given the daily mean and difference in RH exposure. The shaded area shows the 95% CI, reflecting the precision of the FEV_1_ estimate. Covariates adjusted for in the models were age, sex, smoking, and body mass index (BMI). Asterisks (*) mark statistically significant associations where *p<0.05*

**Figure S13.** Associations of odds ratio (OR) for chronic obstructive pulmonary disease (COPD) with particulate matter of an aerodynamic diameter of <2.5 μm (PM_2.5_) were analyzed, considering the joint effect by quartiles of temperature among males. Two-factor models were fitted for the OR for COPD, with four degrees of freedom restricted, and adjusted for age, sex, body mass index, and smoking status. The temperature was divided into quartiles: first quartile (Q_1_), second quartile (Q_2_), third quartile (Q_3_), and fourth quartile (Q_4_), and joined into the PM_2.5_ analysis using the GAMLSS model to assess their joint effects on COPD risk. Asterisks (*) mark statistically significant associations where *p < 0.05*

**Figure S14.** Associations of odds ratio (OR) for chronic obstructive pulmonary disease (COPD) with particulate matter of an aerodynamic diameter of <2.5 μm (PM_2.5_) were analyzed, considering the joint effect by quartiles of temperature among females. Two-factor models were fitted for the OR for COPD, with four degrees of freedom restricted, and adjusted for age, sex, body mass index, and smoking status. The temperature was divided into quartiles: first quartile (Q_1_), second quartile (Q_2_), third quartile (Q_3_), and fourth quartile (Q_4_), and joined into the PM_2.5_ analysis using the GAMLSS model to assess their joint effects on COPD risk. Asterisks (*) mark statistically significant associations where *p < 0.05*

**Figure S15.** Associations of FEV_1_/FVC with particulate matter of an aerodynamic diameter of <2.5 μm (PM_2.5_) were analyzed, considering the joint effect by quartiles of temperature. Two-factor models were fitted for the FEV_1_/FVC, with four degrees of freedom restricted, and adjusted for age, sex, body mass index, and smoking status. The temperature was divided into quartiles: first quartile (Q_1_), second quartile (Q_2_), third quartile (Q_3_), and fourth quartile (Q_4_), and joined into the PM_2.5_ analysis using the GAMLSS model to assess their joint effects on FEV_1_/FVC. Asterisks (*) mark statistically significant associations where *p < 0.05*

**Figure S16.** Associations of FEV_1_ with particulate matter of an aerodynamic diameter of <2.5 μm (PM_2.5_) were analyzed, considering the joint effect by quartiles of temperature. Two-factor models were fitted for the FEV_1_, with four degrees of freedom restricted, and adjusted for age, sex, body mass index, and smoking status. The temperature was divided into quartiles: first quartile (Q_1_), second quartile (Q_2_), third quartile (Q_3_), and fourth quartile (Q_4_), and joined into the PM_2.5_ analysis using the GAMLSS model to assess their joint effects on FEV_1_. Asterisks (*) mark statistically significant associations where *p < 0.05*

**Figure S17.** Associations of FVC with particulate matter of an aerodynamic diameter of <2.5 μm (PM_2.5_) were analyzed, considering the joint effect by quartiles of temperature. Two-factor models were fitted for the FVC, with four degrees of freedom restricted, and adjusted for age, sex, body mass index, and smoking status. The temperature was divided into quartiles: first quartile (Q_1_), second quartile (Q_2_), third quartile (Q_3_), and fourth quartile (Q_4_), and joined into the PM_2.5_ analysis using the GAMLSS model to assess their joint effects on FVC. Asterisks (*) mark statistically significant associations where *p < 0.05*

**Figure S18.** Associations of odds ratio (OR) of chronic obstructive pulmonary disease (COPD)with particulate matter of an aerodynamic diameter of <2.5 μm (PM_2.5_) were analyzed, considering the joint effect by quartiles of relative humidity (RH) among males. Two-factor models were fitted for the OR for COPD, with four degrees of freedom restricted, and adjusted for age, sex, body mass index, and smoking status. The RH was divided into quartiles: first quartile (Q_1_), second quartile (Q_2_), third quartile (Q_3_), and fourth quartile (Q_4_), and joined into the PM_2.5_ analysis using the GAMLSS model to assess their joint effects on COPD risk. Asterisks (*) mark statistically significant associations where *p < 0.05*

**Figure S19.** Associations of odds ratio (OR) of chronic obstructive pulmonary disease (COPD)with particulate matter of an aerodynamic diameter of <2.5 μm (PM_2.5_) were analyzed, considering the joint effect by quartiles of relative humidity (RH) among females. Two-factor models were fitted for the OR for COPD, with four degrees of freedom restricted, and adjusted for age, sex, body mass index, and smoking status. The RH was divided into quartiles: first quartile (Q_1_), second quartile (Q_2_), third quartile (Q_3_), and fourth quartile (Q_4_), and joined into the PM_2.5_ analysis using the GAMLSS model to assess their joint effects on COPD risk. Asterisks (*) mark statistically significant associations where *p < 0.05*

**Figure S20.** Associations of FEV_1_/FVC with particulate matter of an aerodynamic diameter of <2.5 μm (PM_2.5_) were analyzed, considering the joint effect by quartiles of RH. Two-factor models were fitted for the FEV_1_/FVC, with four degrees of freedom restricted, and adjusted for age, sex, body mass index, and smoking status. The RH was divided into quartiles: first quartile (Q_1_), second quartile (Q_2_), third quartile (Q_3_), and fourth quartile (Q_4_), and joined into the PM_2.5_ analysis using the GAMLSS model to assess their joint effects on FEV_1_/FVC. Asterisks (*) mark statistically significant associations where *p < 0.05*

**Figure S21.** Associations of FEV_1_ with particulate matter of an aerodynamic diameter of <2.5 μm (PM_2.5_) were analyzed, considering the joint effect by quartiles of RH. Two-factor models were fitted for the FEV_1_, with four degrees of freedom restricted, and adjusted for age, sex, body mass index, and smoking status. The RH was divided into quartiles: first quartile (Q_1_), second quartile (Q_2_), third quartile (Q_3_), and fourth quartile (Q_4_), and joined into the PM_2.5_ analysis using the GAMLSS model to assess their joint effects on FEV_1_. Asterisks (*) mark statistically significant associations where *p < 0.05*

**Figure S22.** Associations of FVC with particulate matter of an aerodynamic diameter of <2.5 μm (PM_2.5_) were analyzed, considering the joint effect by quartiles of RH. Two-factor models were fitted for the FVC, with four degrees of freedom restricted, and adjusted for age, sex, body mass index, and smoking status. The RH was divided into quartiles: first quartile (Q_1_), second quartile (Q_2_), third quartile (Q_3_), and fourth quartile (Q_4_), and joined into the PM_2.5_ analysis using the GAMLSS model to assess their joint effects on FVC. Asterisks (*) mark statistically significant associations where *p < 0.05*
